# Human genetic basis of coronavirus disease 2019

**DOI:** 10.1038/s41392-021-00736-8

**Published:** 2021-09-20

**Authors:** Hao Deng, Xue Yan, Lamei Yuan

**Affiliations:** 1 Health Management Center, the Third Xiangya Hospital, Central South University, Changsha, China; 2 Center for Experimental Medicine, the Third Xiangya Hospital, Central South University, Changsha, China; 3 Disease Genome Research Center, Central South University, Changsha, China; 4 Department of Neurology, the Third Xiangya Hospital, Central South University, Changsha, China

**Keywords:** Infectious diseases, Predictive markers, Medical genetics

## Abstract

Coronavirus disease 2019 (COVID-19) caused by a novel coronavirus, severe acute respiratory syndrome coronavirus 2 (SARS-CoV-2), has resulted in considerable morbidity and mortality worldwide. COVID-19 incidence, severity, and mortality rates differ greatly between populations, genders, ABO blood groups, human leukocyte antigen (HLA) genotypes, ethnic groups, and geographic backgrounds. This highly heterogeneous SARS-CoV-2 infection is multifactorial. Host genetic factors such as variants in the angiotensin-converting enzyme gene (*ACE*), the angiotensin-converting enzyme 2 gene (*ACE2*), the transmembrane protease serine 2 gene (*TMPRSS2*), along with HLA genotype, and ABO blood group help to explain individual susceptibility, severity, and outcomes of COVID-19. This review is focused on COVID-19 clinical and viral characteristics, pathogenesis, and genetic findings, with particular attention on genetic diversity and variants. The human genetic basis could provide scientific bases for disease prediction and targeted therapy to address the COVID-19 scourge.

## Introduction

Coronavirus disease 2019 (COVID-19) is this century’s third plague and was declared as the sixth international concerned public health emergency by the World Health Organization (WHO) on 30 January 2020.^1,2^ The responsible pathogen is a previously unknown RNA coronavirus.^1,3,4^ It was designated as severe acute respiratory syndrome coronavirus 2 (SARS-CoV-2) by the International Committee on Taxonomy of Viruses.^5^ As of 17 May 2021, the COVID-19 pandemic has resulted in 162,494,817 cases and 3,494,424 deaths worldwide (https://covid19.who.int/). COVID-19 is highly heterogeneous and its severity may relate to multiple factors including health care, quarantine effectiveness, governmental policies, societal norms of behavior, economics, cultural practices, climate, pollution, and viral characteristics, as well as host-associated factors.^6–10^ In the aspect of host-associated factors, in addition to age (>60 years), initial health status, pre-existing diseases, smoking history, and previous vaccinations, individual genetic basis contributes to individual susceptibility, severity, and outcomes of COVID-19.^7,11,12^ Classical twin studies indicated 31% heritability for predicted COVID-19.^13^ Human genetic basis may implicate in significant diversities of COVID-19 among populations with different genders, ABO blood groups, human leukocyte antigen (HLA) genotypes, ethnic groups, and geographic backgrounds.^6,14–16^ Several gene variants related to gene expression and protein function changes were reported as explaining the individual susceptibility, severity, and outcomes.^12,17^

**Fig. 1:**
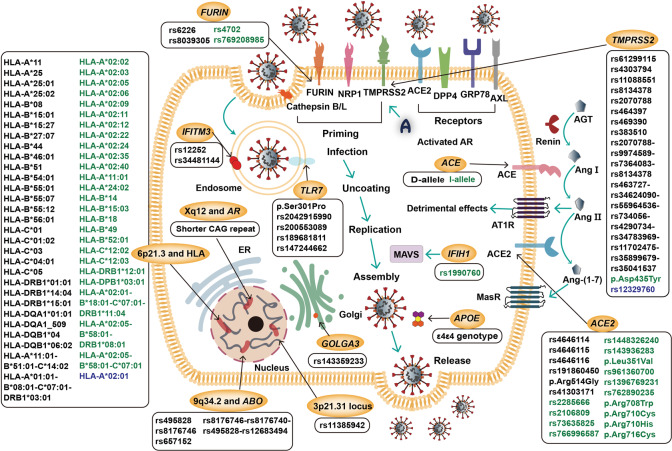
Pathogenesis of SARS-CoV-2 and genetic variants associated with COVID-19. After the recognition of ACE2, DPP4, GRP78, and AXL receptors and the priming by TMPRSS2, FURIN, and NRP1, as well as cathepsin B/L, SARS-CoV-2 enters cells and starts the replication process to assemble and release. Activated AR induces *TMPRSS2* expression. ACE/Ang II/AT1R and ACE2/Ang-(1–7)/MasR axes regulate RAAS to involve in COVID-19. The risk (black), protective (green), and uncertain (blue) variants or alleles or haplotypes for COVID-19 are highlighted. SARS-CoV-2 severe acute respiratory syndrome coronavirus 2, COVID-19 coronavirus disease 2019, ACE2 angiotensin-converting enzyme 2, DPP4 dipeptidyl peptidase 4, GRP78 glucose-regulated protein-78, AXL anexelekto, TMPRSS2 transmembrane protease serine 2, FURIN furin, paired basic amino acid-cleaving enzyme, NRP1 neuropilin-1, AR androgen receptor, AGT angiotensinogen, Ang angiotensin, ACE angiotensin-converting enzyme, AT1R angiotensin II type 1 receptor, MasR Mas receptor, *TLR7* the Toll-like receptor 7 gene, *IFITM3* the interferon induced transmembrane protein 3 gene, HLA human leukocyte antigen, *GOLGA3* the golgin A3 gene, *ABO* the ABO, alpha 1–3-*N*-acetylgalactosaminyltransferase and alpha 1–3-galactosyltransferase gene, *APOE* the apolipoprotein E gene, *IFIH1* the interferon induced with helicase C domain 1 gene, MAVS mitochondrial antiviral signaling protein, ER endoplasmic reticulum

In this review, clinical and viral characteristics, pathogenesis, and the human genetic basis associated with COVID-19 are investigated. Focus is on the protective and risk effects of variants in related genes such as the angiotensin-converting enzyme gene (*ACE*), the angiotensin-converting enzyme 2 gene (*ACE2*), the transmembrane protease serine 2 gene (*TMPRSS2*), and ABO blood groups and HLA genotypes (Table 1 and Fig. 1).

**Table 1:** Summary of human genes associated with COVID-19

Locus	Gene(s) or genotype	Variant(s) or allele(s) or haplotype(s)			References
		Risk	Protective	Uncertain	
2q24.2	*IFIH1*	/	rs1990760	/	^102,103^
3p21.31	*SLC6A20*	/	/	/	^104–108^
	*LZTFL1*	rs11385942	/	/	
	*FYCO1*	/	/	/	
	*CXCR6*	/	/	/	
	*XCR1*	/	/	/	
	*CCR9*	/	/	/	
6p21.3	HLA	HLA-A*11	HLA-A*02:02	HLA-A*02:01	^9,15,16,18,97,115,117–127,130^
		HLA-A*25	HLA-A*02:03		
		HLA-A*25:01	HLA-A*02:05		
		HLA-A*25:02	HLA-A*02:06		
		HLA-B*08	HLA-A*02:09		
		HLA-B*15:01	HLA-A*02:11		
		HLA-B*15:27	HLA-A*02:12		
		HLA-B*27:07	HLA-A*02:22		
		HLA-B*44	HLA-A*02:24		
		HLA-B*46:01	HLA-A*02:35		
		HLA-B*51	HLA-A*02:40		
		HLA-B*54:01	HLA-A*11:01		
		HLA-B*55:01	HLA-A*24:02		
		HLA-B*55:07	HLA-B*14		
		HLA-B*55:12	HLA-B*15:03		
		HLA-B*56:01	HLA-B*18		
		HLA-C*01	HLA-B*49		
		HLA-C*01:02	HLA-B*52:01		
		HLA-C*03	HLA-C*12:02		
		HLA-C*04:01	HLA-C*12:03		
		HLA-C*05	HLA-DRB1*12:01		
		HLA-DRB1*01:01	HLA-DPB1*03:01		
		HLA-DRB1*14:04	HLA-A*02:01-B*18:01-C*07:01-DRB1*11:04		
		HLA-DRB1*15:01	HLA-A*02:05-B*58:01-DRB1*08:01		
		HLA-DQA1*01:01	HLA-A*02:05-B*58:01-C*07:01		
		HLA-DQA1_509			
		HLA-DQB1*04			
		HLA-DQB1*06:02			
		HLA-A*11:01-B*51:01-C*14:02			
		HLA-A*01:01-B*08:01-C*07:01-DRB1*03:01			
9q34.2	*ABO*	rs495828	/	/	^104,135,137–139^
		rs8176746			
		rs657152			
		rs8176746–rs8176740–rs495828–rs12683493			
9q34.3	*DPP7*	1-bp insertion	/	/	^106^
11p15.5	*IFITM3*	rs12252	/	/	^141–146^
		rs34481144			
12q24.33	*GOLGA3*	rs143359233	/	/	^106^
13q12.3	*HMGB1*	/	/	/	^149,150^
15q26.1	*FURIN*	rs6226	rs4702	/	^20,70,151^
		rs8039305	rs769208985		
17q23.3	*ACE*	D-allele	I-allele	/	^80,86,154–158^
19q13.32	*APOE*	ε4ε4 genotype	/	/	^160–163^
21q22.3	*TMPRSS2*	rs61299115	p.Asp435Tyr	rs12329760	^106,165–171^
		rs4303794			
		rs11088551			
		rs8134378			
		rs2070788			
		rs464397			
		rs469390			
		rs383510			
		rs2070788–rs9974589–rs7364083–rs8134378			
		rs463727–rs34624090–rs55964536–rs734056–rs4290734–-rs34783969–rs11702475–rs35899679–rs35041537			
Xp22.2	*TLR7*	p.Ser301Pro	/	/	^181,182^
		rs2042915990			
		rs200553089			
		rs189681811			
		rs147244662			
Xp22.22	*ACE2*	rs4646114	rs2285666	/	^4,8,17,151,152,165,166,168,171,183,184,191–197^
		rs4646115	rs2106809		
		rs4646116	rs73635825		
		rs191860450	rs766996587		
		p.Arg514Gly	rs1448326240		
		rs41303171	rs143936283		
			p.Leu351Val		
			rs961360700		
			rs1396769231		
			rs762890235		
			p.Arg708Trp		
			p.Arg710Cys		
			p.Arg710His		
			p.Arg716Cys		
Xq12	*AR*	Shorter CAG repeat	/	/	^101,176,200–203^

*IFIH1* the interferon induced with helicase C domain 1 gene, *SLC6A20* the solute carrier protein family 6 member 20 gene, *LZTFL1* the leucine zipper transcription factor like 1 gene, *FYCO1* the FYVE and coiled-coil domain autophagy adaptor 1 gene, *CXCR6* the C-X-C motif chemokine receptor 6 gene, *XCR1* the X-C motif chemokine receptor 1 gene, *CCR9* the C-C motif chemokine receptor 9 gene, HLA human leukocyte antigen, *ABO* the ABO, alpha 1–3-*N*-acetylgalactosaminyltransferase and alpha 1–3-galactosyltransferase gene, *DPP7* the dipeptidyl peptidase 7 gene, *IFITM3* the interferon induced transmembrane protein 3 gene, *GOLGA3* the golgin A3 gene, *HMGB1* the high-mobility group box 1 gene, *FURIN* the furin, paired basic amino acid cleaving enzyme gene, *ACE* the angiotensin-converting enzyme gene, *APOE* the apolipoprotein E gene, *TMPRSS2* the transmembrane protease serine 2 gene, *TLR7* the Toll-like receptor 7 gene, *ACE2* the angiotensin-converting enzyme 2 gene, *AR* the androgen receptor gene.

## Clinical characteristics

**Fig. 2:**
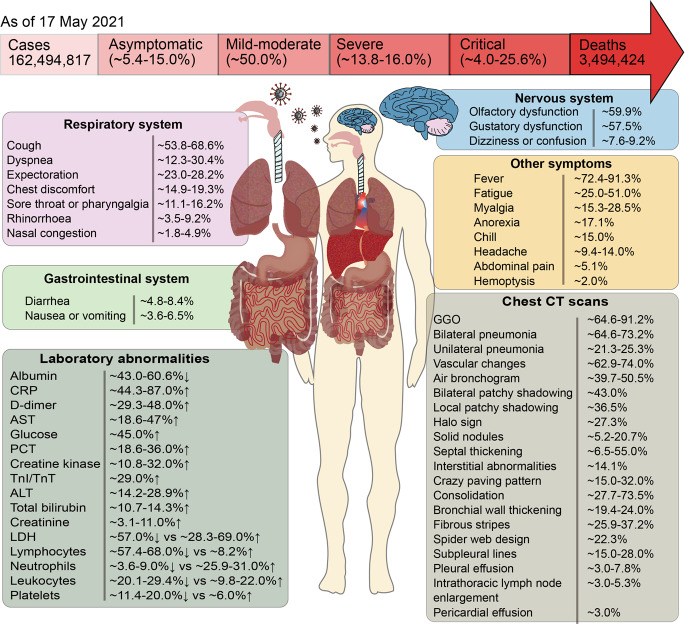
Overview of clinical characteristics of COVID-19. CRP C-reactive protein, AST aspartate aminotransferase, PCT procalcitonin, TnI/TnT troponin I/troponin T, ALT alanine aminotransferase, LDH lactate dehydrogenase, CT computed tomography, GGO ground-glass opacity

The COVID-19 clinical spectrum is heterogeneous (Fig. 2) and ranges from asymptomatic (∼5.4–15.0%), mild–moderate (∼50.0%), severe (∼13.8–16.0%) to critical (∼4.0–25.6%) status.^18–22^ Typical symptoms in most patients are mild and nonspecific, including fever (∼72.4–91.3%), cough (∼53.8–68.6%), smell dysfunction (∼59.9%), taste dysfunction (∼57.5%), fatigue (∼25.0–51.0%), dyspnea (∼12.3–30.4%), myalgia (∼15.3–28.5%), expectoration (∼23.0–28.2%), chest discomfort (∼14.9–19.3%), anorexia (∼17.1%), sore throat or pharyngalgia (∼11.1–16.2%), chill (∼15.0%), headache (∼9.4–14.0%), dizziness or confusion (∼7.6–9.2%), rhinorrhoea (∼3.5–9.2%), diarrhea (∼4.8–8.4%), nausea or vomiting (∼3.6–6.5%), abdominal pain (∼5.1%), nasal congestion (∼1.8–4.9%), and hemoptysis (∼2.0%).^2,3,19,21–31^ Some patients (usually those with advanced age and comorbidities) may rapidly progress to viral pneumonia, life-threatening acute respiratory distress syndrome, or multiple organ failures.^32,33^ In addition to the respiratory tract and lungs being primarily affected, other organs and systems such as the heart, blood vessels, gastrointestinal tract, liver, kidneys, skin, and nervous systems, can be adversely involved.^34,35^ Common laboratory abnormalities in COVID-19 patients are decreased albumin (∼43.0–60.6%), increased C-reactive protein (∼44.3–87.0%), D-dimer (∼29.3–48.0%), aspartate aminotransferase (∼18.6–47.0%), glucose (∼45.0%), procalcitonin (∼18.6–36.0%), creatine kinase (∼10.8–32.0%), troponin I/troponin T (∼29.0%), alanine aminotransferase (∼14.2–28.9%), total bilirubin (∼10.7–14.3%), and creatinine (∼3.1–11.0%), and decreased or increased lactate dehydrogenase (∼57.0% ↓ vs ∼28.3–69.0% ↑), lymphocytes (∼57.4–68.0% ↓ vs ∼8.2% ↑), neutrophils (∼3.6–9.0% ↓ vs ∼25.9–31.0% ↑), leukocytes (∼20.1–29.4% ↓ vs ∼9.8–22.0% ↑), and platelets (∼11.4–20.0% ↓ vs ∼6.0% ↑).^25,28,29,36^ Chest computed tomography (CT) scans revealed ground-glass opacity (∼64.6–91.2%), lesions consistent with bilateral (∼64.6–73.2%) or unilateral (∼21.3–25.3%) pneumonia, vascular changes (∼62.9–74.0%), air bronchogram (∼39.7–50.5%), bilateral or local patchy shadowing (∼43.0%, ~36.5%), halo sign (∼27.3%), solid nodules (∼5.2–20.7%), septal thickening (∼6.5–55.0%), interstitial abnormalities (∼14.1%), crazy paving pattern (∼15.0–32.0%), consolidation (∼27.7–73.5%), bronchial wall thickening (∼19.4–24.0%), fibrous stripes (∼25.9–37.2%), spider web design (∼22.3%), subpleural lines (∼15.0–28.0%), pleural effusion (∼3.0–7.8%), intrathoracic lymph node enlargement (∼3.0–5.3%), and pericardial effusion (∼3.0%).^21,22,25,37–40^

## The causative pathogen, SARS-CoV-2

**Fig. 3:**
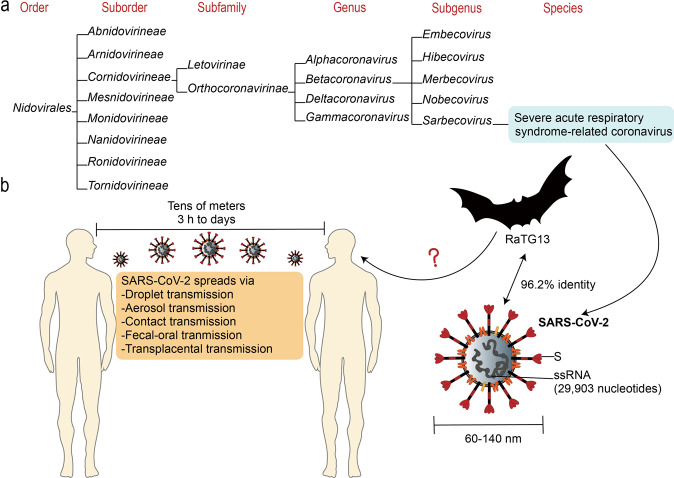
The causative pathogen, SARS-CoV-2. **a** The taxonomy of SARS-CoV-2 is shown (from https://talk.ictvonline.org/taxonomy/). **b** SARS-CoV-2 is ssRNA virus with a diameter of ~60–140 nm and whole viral genome sequence of 29,903 nucleotides, and possesses distinctive S protein; 96.2% identity is shown between SARS-CoV-2 and a bat-borne coronavirus, RaTG13. SARS-CoV-2 spreads from human to human via droplet, aerosol, contact, fecal–oral, and transplacental transmissions. Droplets with SARS-CoV-2 can spread up to tens of meters and remain viable and infectious for 3 h to days. SARS-CoV-2 severe acute respiratory coronavirus 2, S spike protein, ssRNA single-strand RNA

SARS-CoV-2 is an enveloped, non-segmented, positive-sense single-stranded RNA virus with 29,903 nucleotides in its genome sequence containing 5′ capped and 3′ polyadenylated.^41–43^ The 5′ two-thirds region of the genome is occupied by two large open reading frames (ORFs), ORF1a and ORF1b, that encodes 15–16 nonstructural proteins.^44^ Other functional ORFs encode structural and accessory proteins.^45^ The structural proteins include the distinctive spike (S), envelope (E), membrane (M), and nucleocapsid (N) proteins, among which the S, E, and M proteins compose the envelope structure, while the N protein encapsulates the viral genome.^46^ SARS-CoV-2 is generally spherical with some pleomorphism and a diameter of ∼60–140 nm.^3^ The viral-enveloped lipid bilayer consists of cholesterols and phospholipids, which makes the virus susceptible to dry heat, detergents, and organic solvents.^46^ This novel coronavirus was assigned to the genus *Betacoronavirus* in the family *Coronaviridae* of the order *Nidovirales* (Fig. 3).^18,42,47^ SARS-CoV-2 has a 96.2% identity throughout the genome to RaTG13, a bat-borne coronavirus in *Rhinolophus affinis.*^48^ Droplet, aerosol, contact, fecal–oral and transplacental transmissions are documented human-to-human transmission routes.^49–53^ Small droplets with SARS-CoV-2 can travel tens of meters in favorable atmospheric conditions and remain viable and infectious from 3 h to days.^32,54,55^ Patients mildly affected and asymptomatic carriers constituting the majority of COVID-19 cases are thought to be primarily responsible for the spread of SARS-CoV-2.^10,56^

As of 17 May 2021, the worldwide circulating SARS-CoV-2 variants mainly include the B.1.1.7 (63%), B.1.617.2 (22%), P.1 (6%), B.1.526 (2%), and others (https://covid19dashboard.regeneron.com). Lineage B.1.1.7 first detected in the United Kingdom in September 2020 has 21 characteristic mutations and exists in comparative transmission effectivity.^57^ Among the S protein mutations, the N501Y substitution would change the receptor-binding domain conformation and may slightly increase 18% of fatality risk. Mutation del69–70, considered to be responsible for stronger viral transmissibility, may cause S-gene target failure and produce a positive result for other targets at real-time reverse transcription-polymerase chain reaction assays, which could be a proxy for diagnosing B.1.1.7 infections.^58,59^ Other two S protein substitutions, E484K in some strains of B.1.1.7 and L452R in B.1.617.2, may lead to much poorer effectivity of specific monoclonal antibody treatment in the corresponding infected cases (https://www.cdc.gov).

## Pathogenesis of SARS-CoV-2

### Entry and replication of SARS-CoV-2

Cell entry of SARS-CoV-2 depends on two determinants: (1) the viral S protein recognizes ACE2 receptor and (2) TMPRSS2 primes S protein.^60,61^ The S1 subunit of the envelope-embedded S glycoprotein attaches to the cellular ACE2 receptor via the polar contacts of hydrophilic residues.^60,62^ TMPRSS2 may trigger a proteolytic cleavage at the S1/S2 multibasic cleavage site.^63,64^ Other than ACE2, some studies reported that the human dipeptidyl peptidase 4 (DPP4), the cell-surface glucose-regulated protein-78, and the receptor tyrosine kinase anexelekto may also be conducive to viral entry and infection.^65–68^ Cathepsin B/L and furin, paired basic amino acid cleaving enzyme (FURIN) may also catalyze S protein proteolytic cleavage.^64,69^ As the S protein is cleaved to the S1 and the S2 domain, the cell surface receptor neuropilin-1 (NRP1) may bind to the C-terminal functional furin-cleavage sequence of the S1 domain more strongly in some cases and help the S2 isolating from the S1 domain.^70,71^ After S1 detachment, the S2 subunit undergoes a conformational change and mediates the fusion between the virus and the host membranes to mediate viral infection.^71,72^ The virus enters the cytoplasm and starts the replication process to assemble new viral particles and amplify its viral load.^69,73^ During the viral entry and replication process, cyclophilin A is essential for viral replication and its interaction with CD147 may mediate SARS-CoV-2 entering the host cells.^65^

### Renin–angiotensin–aldosterone system (RAAS)

RAAS is a complex system involved in multiple biological processes that are responsible for inducing a cascade of vasoactive peptides, which regulate vascular and renal functions.^54,74,75^ In the blood, the precursor angiotensinogen (AGT) is hydrolyzed to angiotensin I (Ang I) by the active renin.^76,77^ In the lungs, ACE removes the C-terminal dipeptide of Ang I to produce a potent vasoconstrictor, Ang II, which promotes detrimental effects by acting on Ang II type 1 receptor (AT1R).^78–80^ ACE2 removes a single C-terminal amino acid from Ang I and Ang II to generate Ang-(1–9) and Ang-(1–7), of which the former is in turn converted to Ang-(1–7) by ACE.^81^ Ang-(1–7) counters Ang II cellular and molecular effects by binding and activating the G-protein-coupled Mas receptor (MasR).^79,82,83^ ACE2 catalytic efficiency with Ang II as a substrate is 400-fold higher than with Ang I.^83,84^ Accordingly, ACE2, an ACE homolog, is a key negative regulator antagonizing the activation of the classical RAAS by counterbalancing ACE actions.^82,85^ ACE and ACE2 maintain the homeostasis via the “adverse” ACE/Ang II/AT1R axis and the “protective” ACE2/Ang-(1–7)/MasR axis. In general, ACE/ACE2 imbalance contributes to RAAS overactivation and pulmonary shutdown, and a high ACE activity and a reduced *ACE2* expression would increase the risk of pulmonary and cardiovascular diseases.^86^ ACE2 mainly binds to cell membranes and rarely exists in a circulating soluble form.^72^ Continued viral infection and replication markedly downregulate ACE2 receptors, leading to the loss of the catalytic effect of membrane ACE2, and thus results in unopposed acute Ang II aggregation and local RAAS activation.^74^ During ACE2 downregulation, an accentuating RAAS imbalance may further exacerbate pathophysiological alteration in COVID-19.^85,87^

### Immunopathogenesis

SARS-CoV-2 activates innate and acquired immune response, and further impairs the immune system and causes cytokine storm, which is an uncontrolled inflammatory response with elevations of circulating cytokine levels.^73,88,89^ The initial antiviral responses are promoted by pattern recognition receptors (PRRs) detecting pathogen-associated molecular patterns (PAMPs).^90^ Retinoic acid-inducible gene I (RIG-I) is an interferon (IFN)-stimulated gene (ISG), and RIG-I-mediated signaling could promote induction of antiviral IFN responses.^90,91^ Recognition of virus promotes downstream transduction in nuclear factor-κB, IFN regulatory factor-3, and Janus kinase-signal transducer and activator of transcription signaling pathways.^92^ Innate immune cells, such as parenchymal cells, neutrophils, dendritic cells, and macrophages, are stimulated to secrete inflammatory mediators.^93^ SARS-CoV-2 disturbs the immune system with its immune evasion strategies, in which viral PAMPs escape from the detection of cytosolic PRRs efficiently.^94^ The virus weakens the antiviral effects of ISG products through dysregulating IFN signaling and IFN generation.^94,95^ In acquired immunity, SARS-CoV-2 may target the CD147 spike protein of T lymphocytes.^96^ Viral peptides are presented by major histocompatibility complex (MHC) Class-I molecules to CD8^+^ T cells (cytotoxic T cells) to kill the virus directly, while by MHC Class-II molecules to CD4^+^ T cells (helper T cells).^96,97^ CD4^+^ T cells generate proinflammatory cytokines and mediators to facilitate other immune cells.^88^ B lymphocytes are directly stimulated by SARS-CoV-2 and interact with CD4^+^ T cells to produce substantial immunoglobulin G antibodies, which lead to the disruption of the virus and increased proinflammatory cytokines.^14,96^ Complement activation through classical or alternative pathways generates a number of chemotactic/inflammatory mediators.^91^ The release of proinflammatory cytokines also could be induced by increased A disintegrin and metalloproteinase 17 activity due to viral invasion.^82^ The cytokine storm, which is characterized by a radical rise in the number of inflammatory cytokines/chemokines such as interleukin-2 (IL-2), IL-6, IL-7, IL-10, granulocyte-colony stimulating factor, IFN-γ-inducible protein 10, tumor necrosis factor-α, macrophage inflammatory protein-1α, monocyte chemoattractant protein-1, C–X–C motif ligand 9 (CXCL9), CXCL10, and CXCL11, triggers extensive tissue injury and body dysfunction and is considered as the primary contribution to mortality in COVID-19.^32,33,97,98^ Among these inflammatory cytokines/chemokines, IL-6 was reported to play a key role in COVID-19 cytokine storm development.^89^ However, a study found that only one cytokine, macrophage migration inhibitory factor, was significantly higher in COVID-19 patients than healthy controls. In addition, elevations of IL-6 were only found in some severe/critical patients and much less than patients with other cytokine storm syndrome-associated diseases.^99^ These controversial results pointed that SARS-CoV-2 causes a chemokine storm, not a cytokine storm, providing an interesting insight into COVID-19 immunopathogenesis.^99^ Genetically determined individual differences in immunity may relate to both variants in the immune-related genes and the inherent differences in the X- and Y-chromosome gene expressions.^100,101^

## Autosomal loci and genes associated with COVID-19

### 2q24.2 and the interferon induced with helicase C domain 1 gene (*IFIH1*)

The IFIH1 protein is a primary PRR that first senses the coronavirus RNA and then triggers innate immunity and activates mitochondrial antiviral signaling protein.^102^ The variant rs1990760 (p.Ala946Thr) of the *IFIH1* gene has been reported to be positively related to increased expression of the viral resistance gene *IFIH1* and IFN-induced gene.^103^ This polymorphic variant in various ethnic populations is correlated with population migration and originated from the European and Asian populations. It is expected that rs1990760 T-allele confers carriers more resistance to COVID-19, e.g., Africans and African-Americans with low-frequency ranging from 0.06 to 0.35 have a more vulnerable risk for COVID-19 than Caucasians and Indians with an overall frequency of 0.56.^102^

### 3p21.31

A genome-wide association study (GWAS) conducted in Italy and Spain revealed that a 3p21.31 gene cluster comprised of the solute carrier protein family 6 member 20 gene (*SLC6A20*), the leucine zipper transcription factor like 1 gene (*LZTFL1*), the FYVE and coiled-coil domain autophagy adaptor 1 gene (*FYCO1*), the C–X–C motif chemokine receptor 6 gene (*CXCR6*), the X–C motif chemokine receptor 1 gene (*XCR1*), and the C-C motif chemokine receptor 9 gene (*CCR9*) is a genetically susceptible locus in severe COVID-19 patients with respiratory failure.^104,105^ The *SLC6A20* gene encodes a transporter, signaling threshold regulating transmembrane adaptor, which functionally interacts with ACE2.^105^ The *CXCR6* gene and the *CCR9* gene encoding chemokine receptors are implicated in T cell differentiation and recruitment.^95^ Rs11385942 in the *LZTFL1* gene associated with increased *SLC6A20* expression and reduced *CXCR6* expression is a risk variant and is common in Europeans, Africans, and South Asians, but almost absent in the East Asians.^106^

Another study suggests that a 49.4 kb haplotype in high linkage disequilibrium (LD) on 3p21.31 is the most highly correlated to severe COVID-19, and this core haplotype is thought to have entered the human population from the Neanderthals, an extinct hominin ~40,000 to 60,000 years ago.^107,108^ Neanderthal-derived core haplotype frequency varies significantly among populations that 63% of the Bengalese, ~30% of the South Asians, 8% of the Europeans, 4% of the Americans, a lower frequency of the East Asians, and almost none of the Africans carry this risk haplotype.^109^ This could explain that Briton-originated studies showed higher mortality in COVID-19 patients of Bangladeshi ethnicity (~2 times higher) and of South Asian descent.^108,110^ Correspondingly, mortality rates reported on 14 July 2020 in South Africa, Japan, South Korea, and China were substantially lower than the Western countries (in North America and West Europe).^111^ However, black individuals were at higher risk compared with white people in England and the United States.^112–114^ This paradoxical fact may be explained by the impacts of other genetic and environmental factors.

### 6p21.3 and HLA genotype

The HLA system containing nearly 27,000 alleles in classes I, II, and III is an exceedingly polymorphic region.^115,116^ Genetic variations across the *HLA*-*A*, *HLA*-*B*, *HLA*-*C*, *HLA*-*DR*, *HLA*-*DP*, and *HLA*-*DQ* genes, which encode MHC molecules, might change the process of viral infection by differentially mediating antiviral immunity.^109,117^ Several studies suggest that there may be specific risk and protective HLA alleles or haplotypes for COVID-19 incidence and mortality.^9^.

HLA-A and HLA-C were reported to have the relatively greatest and least capacities for presenting SARS-CoV-2, respectively, and HLA-B preferentially involves susceptibility to COVID-19.^16,118^ HLA-A*25:01, HLA-A*25:02, HLA-B*46:01, HLA-C*01:02, and HLA-B22 serotype, including HLA-B*54:01, HLA-B*55:01, HLA-B*55:07, HLA-B*55:12, and HLA-B*56:01, are weak presenters, and thus individuals with these alleles may be COVID-19 susceptible.^118–120^ HLA-A*02 subtypes such as HLA-A*02:02, HLA-A*02:03, HLA-A*02:05, HLA-A*02:06, HLA-A*02:09, HLA-A*02:11, HLA-A*02:12, HLA-A*02:22, HLA-A*02:24, HLA-A*02:35, and HLA-A*02:40, as well as HLA-A*24:02, HLA-B*15:03, HLA-B*52:01, HLA-C*12:02, and HLA-C*12:03, are strong presenters for SARS-CoV-2 epitopes and predicted to be protective.^119,121,122^ SARS-CoV-2 peptides presented by HLA-B*15:03 are common among human coronaviruses and enable cross-protective T cell-based immunity.^120^ HLA-A*02:01 has varying capacities for presenting SARS-CoV-2 antigens in different studies.^15,117^

Several studies concluded that HLA-A*25, HLA-B*08, HLA-B*15:01, HLA-B*15:27, HLA-B*27:07, HLA-B*44, HLA-B*51, HLA-C*01, HLA-C*03, HLA-C*04:01, HLA-DRB1*15:01, HLA-DQA1_509, HLA-DQB1*04, and HLA-DQB1*06:02 were associated with higher occurrence and mortality, while HLA-B*14, HLA-B*18, and HLA-B*49 showed an inverse log-linear relationship with COVID-19.^123–127^ HLA-A*11 was positively associated with COVID-19 mortality, but another analysis suggested that HLA-A*11:01 could generate efficient antiviral responses.^115,117^ HLA-DRB1*01:01 (severe 2.2% vs mild 0.5%), HLA-DRB1*14:04 (severe 2.0% vs mild 0.5%), and HLA-DQA1*01:01 (severe 2.9% vs mild 0.9%) are risk alleles for severe COVID-19, while HLA-DRB1*12:01 (severe 2.2% vs mild 3.7%) and HLA-DPB1*03:01 (severe 0.7% vs mild 4.5%) were protective.^106^ HLA-C*05 is significantly correlated to increased COVID-19 death risk and each increase of 1% in HLA-C*05 frequency is followed by an increase of 44 deaths/million. Its receptor KIR2DS4fl is located on natural killer (NK) cells and recognizes viral peptides bound to HLA-C*05 to generate a potent activation signal, leading to NK cell-induced hyperactive antiviral immunity jointly with HLA-C*05.^9^ Several South-East Asian and Oceania regions seem to correspond to higher predicted protective allele frequencies than other global regions based on data from the Allele Frequency Net Database (http://www.allelefrequencies.net/hla.asp; Supplementary Fig. S1, 2). HLA-A*24:02 was found to bind the peptide VYIGDPAQL, which is a virus helicase fragment shared between SARS-CoV-2 and two common cold coronaviruses, human coronavirus OC43 and HKU1. Thus, it was assumed that the anti-VYIGDPAQL T cells primed by previous OC43 or HKU1 infections could be restimulated after SARS-CoV-2 infection.^128^ HLA-A*24:02 allele carried by 25.5–98.0% of Chinese may partly explain the better epidemic prevention effect in China.

HLA is codominant and expresses all the alleles in the high gene density, complex LD, and homology regions.^109,116^ This suggests studying complete HLA genotypes for each individual rather than being limited to a few protective or harmful alleles as a wiser course.^129^ Haplotype HLA-A*11:01-B*51:01-C*14:02 was more common in severe COVID-19 patients than in mild ones.^106^ An Italian study found that haplotype HLA-A*01:01-B*08:01-C*07:01-DRB1*03:01 contributed to COVD-19 higher occurrence and mortality in northern Italy, while haplotype HLA-A*02:01-B*18:01-C*07:01-DRB1*11:04 closely linked to lower occurrence and mortality in central-southern Italy.^130^ A Sardinian study identified two haplotypes HLA-A*02:05-B*58:01-DRB1*08:01 and HLA-A*02:05-B*58:01-C*07:01 as being protective against severe COVID-19.^123^

Since COVID-19 vaccines may have variable binding affinities with different HLA genotypes in different populations, predicting good binders across certain HLA alleles may contribute to design an efficacious COVID-19 vaccine with corresponding epitope targets.^131^

### 9q34.2 and the ABO, alpha 1–3-*N*-acetylgalactosaminyltransferase and alpha 1–3-galactosyltransferase gene (*ABO*)

A, B, and O blood groups possess A-antigen, B-antigen, and the biosynthetic precursor H-antigen, respectively.^132,133^ The antigen-encoding gene comprises A, B, and O alleles and is expressed in four genetic phenotypes.^132^ SARS-CoV-2 susceptibility and survival following infection may relate to ABO blood groups. Individuals carrying blood group A have a higher COVID-19 risk, while blood group O exerts a relatively protective effect.^132,134^ In the blood group A, A-antigen causes more P-selectin and intercellular cell adhesion molecule 1 attached to endothelial cells to increase cardiovascular disease likelihood. Blood group O individuals with ~25% decreased levels of von Willebrand factor might have lower thrombotic disease risk.^134–136^ In the blood group B, natural anti-A antibodies might exert a neutralizing activity blocking adhesion between S proteins and ACE2.^133,134^

The GATC haplotype rs8176746–rs8176740–rs495828–rs12683493, of which position is coincident with *ABO* locus, is common in people with non-O blood groups and positively correlated to ACE activity, while blood group O is characterized by intermediate ACE activity.^135,137^ Variants account for 15% of ACE activity variance, of which rs8176746 and rs495828 may independently reckon 2.8% and 4.9%, respectively.^137–139^ The *ABO* variant rs657152 was considered as a significant signal associating with severe COVID-19 in Italian and Spanish cohorts.^104^

### 9q34.3 and the dipeptidyl peptidase 7 gene (*DPP7*)

A 1-base pair (bp) insertion in the *DPP7* gene destroying *DPP7* transcription may have a potential monogenic effect for asymptomatic COVID-19 in a Chinese family analysis.^106^ DPP7 known as a survival factor to maintain lymphocytes quiescently may potentially involve in COVID-19 immunopathogenesis.^140^ The specific functional effects of the *DPP7* gene in COVID-19 still need further clarification.

### 11p15.5 and the interferon induced transmembrane protein 3 gene (*IFITM3*)

The rs12252 C-allele homozygosity in the *IFITM3* gene relates to COVID-19 patient disease severity, and CC-homozygote patients have a 6.37 times higher risk of severity after a SARS-CoV-2 infection.^141–143^ This association is not thought to stem directly from rs12252, but from a functional variant existing LD with rs12252 of *IFITM3* or a nearby gene.^144^ Rs34481144 A-allele (38–56% in Europeans, 2–14% in Africans, and 1–2% in Chinese) might increase COVID-19 susceptibility by triggering methylation of the *IFITM3* promoter to decrease *IFITM3* mRNA expression in CD8^+^ T cells and depressing surrounding gene transcription.^145,146^

### 12q24.33 and the golgin A3 gene (*GOLGA3*)

Pedigree analysis of Chinese suggested the splice acceptor variant rs143359233 in the *GOLGA3* gene potentially implicated in critically ill COVID-19 patients as a monogenic factor.^106^ The *GOLGA3* gene encodes a Golgi complex-associated protein, which participates in protein transportation, cell apoptosis, Golgi positioning, and spermatogenesis.^147^ Its defect was proved to lead to male infertility previously, but the reliable relationship between the *GOLGA3* gene and COVID-19 remains uncertain.^147^ *GOLGA3* may implicate COVID-19 severity by influencing the interaction of SARS-CoV-2 to innate immune pathways.^148^

### 13q12.3 and the high mobility group box 1 gene (*HMGB1*)

The *HMGB1* gene encodes a DNA-binding protein, which is a critical damage-associated molecular pattern (DAMP) and probably regulates a proviral gene expression program. HMGB1 may interact with Toll-like receptor 4 (TLR4) and the advanced glycosylation end-product specific receptor to induce cytokine storm in immune cells and *ACE2* expression in alveolar epithelial cells, further increasing COVID-19 susceptibility.^149,150^

### 15q26.1 and the *FURIN* gene

The *FURIN* gene encodes a ubiquitous membrane-bound pro-protein convertase that cleaves the SARS-CoV-2 S protein into the S1 and S2 subunits. Two highly frequent *FURIN* variants relating to upregulated FURIN in Africans, rs6226 (93%) and rs8039305 (81%), are associated with increased hypertension risk and SARS-CoV-2 infection.^151^ A common variant, rs4702, may directly reduce SARS-CoV-2 infection. The variant rs769208985 (p.Arg298Gln), representing glutamine residue by replacing arginine in a highly conserved position (R298), might influence FURIN recognition of the SARS-CoV-2 S protein.^20,70^

### 17q23.3 and the *ACE* gene

The insertion of an Alu repeat element into *ACE* intron 16 may result in alternative splicing in which the *ACE* I-allele leads to protein shortening and the loss of a catalytically active protein domain, while the *ACE* D-allele still maintains two active protein domains catalyzing Ang I to Ang II.^152,153^ Approximately 60% of ACE level variability in general populations is likely to be determined by the *ACE* I/D variant.^154,155^ The I/D variant is associated with ACE circulating and tissue concentrations, which means that ACE activity levels in I/I carriers are about half of that of D/D carriers.^87,156^ COVID-19 variable recovery and prevalence rates correlate to the ratio of the *ACE* I/D allele frequency and the geographical variations of the *ACE* I/D variant.^157,158^

The racial difference in the *ACE* gene polymorphism is well understood. According to the “thrifty genotype” hypothesis put forward by J.V. Neel, after modern human ancestors expanded out of Africa ~200,000 years ago, genetic variation of the D-allele occurred as the D-allele favoring the retention of salt and water became detrimental.^159^ Middle Eastern populations, particularly those in Lebanon with a relatively low I-allele frequency, are believed to be the ancestor of the *ACE* variant.^86^ I/I genotype increases westwards and eastwards from the Middle East. The distribution of D-allele is characterized by the highest frequency of D-allele in Africa and Arab regions, medium frequencies in Europe, Australia, and America, and the lowest frequency in East Asia.^159^ Therefore, the higher recovery rate in East Asians and disproportionately higher fatality rate in African Americans are unsurprising.^155,158^

### 19q13.32 and the apolipoprotein E gene (*APOE*)

The *APOE* gene has three common alleles, ε2, ε3, and ε4, which are haplotypes of rs429358 and rs7412.^160,161^ Compared to the most common *APOE* ε3ε3 genotype, individuals who are homozygous for *APOE* ε4 have twice the risk of severe COVID-19, although mortalities between *APOE* ε3ε4 and ε3ε3 COVID-19-positive subjects have no significant difference.^162,163^ The *APOE* ε4ε4 homozygous genotype might have a higher risk of severe COVID-19 due to regulating proinflammatory pathways and lipoprotein function being affected.^161,163^ An African-American ε4-allele frequency of 29.5% compared to a Caucasian rate of 12.1% may explain the diverse mortalities.^162^

### 21q22.3 and the *TMPRSS2* gene

The *TMPRSS2* gene variants may play a significant role in the interindividual differences particularly in the gender-related bias of COVID-19 susceptibility and severity.^164^ Rs61299115, rs4303794, and rs11088551 have relatively high frequencies in the general populations (25–36%), but much lower, 2%, in East Asian populations. They potentially enhance *TMPRSS2* transcription, and thus the rarity of these three single-nucleotide variants (SNVs) among the East Asians results in lower *TMPRSS2* expression levels.^165^ Rs12329760 (p.Val197Met) located at the exonic splicing enhancer site might considerably increase the *TMPRSS2* faulty expression, weaken TMPRSS2 stability, and inhibit S protein and ACE2 interaction, which may contribute to asymptomatic and mild patients in Chinese with higher variant frequency.^166^ However, in Italian populations, rs12329760, as well as a haplotype rs2070788–rs9974589–rs7364083–rs8134378, trigger increased *TMPRSS2* expression and may explain the higher mortality rate among the Italians with higher variant frequency.^167^ Rs8134378 close to an androgen-responsive enhancer possibly increases the *TMPRSS2* gene expression in males in an androgen-specific manner and is co-regulated with a “European” haplotype rs463727–rs34624090–rs55964536–rs734056–rs4290734–rs34783969–rs11702475–rs35899679–rs35041537.^168^ The variants rs2070788, rs464397, rs469390 (p.Val379Ile), and rs383510 could upregulate *TMPRSS2* expression in lung tissue and have lower frequencies in East Asians than Africans, Europeans, and Americans, which might explain the different COVID-19 susceptibilities in different populations.^169,170^ Conversely, p.Asp435Tyr only presenting at a low frequency in Europeans leads to the lack of a key residue catalyzing substrate binding.^171^

## X- or Y-linked loci and genes associated with COVID-19

Consistent with Lyon’s theory, X-chromosome inactivation (XCI), which occurs in females in the late blastocyst stage, is a fundamental event in the epigenetic gene regulation that one of the X chromosomes is stochastically inactivated to equal X-linked gene dosage between genders.^167,172–174^ Two noncoding RNAs control this complex inactivation process, which condenses one X chromosome into a compact structure, Barr body, and maintains an active X chromosome simultaneously. Approximately 15–30% of X-linked genes, most are on the short arm (p), can escape from the XCI.^172,175^ Interestingly, XCI is cell-specific such that some cells express the maternal copy, while others express the paternal copy, and escape from XCI can be variable between individuals, among cells in a tissue, and during growth and aging.^176,177^ The skewed XCI may bypass the deleterious X-linked variants in females, while any abnormal gene variants on the X chromosome of males are more likely to express phenotypically and to cause more pronounced consequences due to hemizygosity.^160,175,178,179^ This appears to explain SARS-CoV-2 infection rate gender bias.

### Xp22.2 and the *TLR7* gene

The *TLR7* gene encodes a Toll-like receptor that could recognize SARS-CoV-2 RNA and trigger the antiviral response.^180^ An analysis performed on two young brother pairs with severe COVID-19 identified a maternally inherited variant rs2042915990 (p.Gln710Argfs*18) and a missense variant rs200553089 (p.Val795Phe) as rare loss-of-function (LOF) variants in the *TLR7* gene, which result in immunodeficiencies in type I and II interferon responses.^181^ Further, a nested case–control study identified the *TLR7* gene variants p.Ser301Pro, rs189681811 (p.Arg920Lys), and rs147244662 (p.Ala1032Thr) as LOF variants, which in young, male, severe COVID-19 patients were considered to account for COVID-19 susceptibility in up to 2% cases.^182^

### Xp22.22 and the *ACE2* gene

The *ACE2* gene encoding a dipeptidyl carboxydipeptidase with 805 amino acids is a putative risk factor for SARS-CoV-2 infection.^72,183^ ACE2 contains a potential N-terminal signal peptide, a peptidase domain, and a C-terminal collectrin-like domain, which ends with the single transmembrane helix. A ferredoxin-like fold “Neck” domain is between the peptidase domain and transmembrane helix. The crucial roles of the peptidase and neck domains (residues 19–726) in the ACE2 homodimerization allow for positing that variants affecting these amino acid residues may influence viral infection.^184–187^

Up to the date of this writing, no genetically monogenic, naturally resistant *ACE2* mutations which counter S protein binding have been reported.^188^ However, a number of *ACE2* variants may influence COVID-19 susceptibility and outcomes via three primary routines: (1) alterations of ACE2-binding properties to sirtuin 1, which regulates transcriptional and post-translational modifications of the *ACE2* gene, (2) alteration of the soluble ACE2 levels in circulation and the affinity and density of ACE2 for the S protein, and (3) alteration of circulating Ang-(1–7), which causes a greater marked RAAS imbalance and greater disease severity.^80,87,185,189^

Among the most significant variants for ACE2 activity and levels, the most frequent is the transition rs2285666 (G8790A).^152,190^ The A-allele carriers may have higher serum ACE2 levels than the G-allele carriers, of which A/A genotype had almost 50% higher *ACE2* expression levels than the G/G genotype.^191,192^ Rs2285666 located in the intronic-consensus splice site region might theoretically affect the processing of total RNA to mRNA with alternative splicing mechanisms and further the amount of protein.^193^ The transition G8790A was predicted to lead to ~9.2% increased strength of the splice site and further elevated ACE2 serum levels.^192^ Accordingly, rs2285666 is suggested to be a protective variant to COVID-19, and lower morbidity and mortality in Indians could be explained by the A-allele of rs2285666.^193^ The variant rs2106809 reported in Indians and Saudi Arabians may primarily influence serum ACE2 levels, and that the C/C or C/T genotype has comparatively higher levels than the T/T genotype.^166^

Rs4646114 and rs4646115, which are more prevalent in African descent populations with frequencies of 5.0–7.2% and 1.4–1.8%, could accelerate viral infection and spread, and thus may associate with higher COVID-19 susceptibility.^165^ Rs4646116 (p.Lys26Arg), which is quite frequent in Caucasians, but has not yet been detected in the East-Asian populations, activates ACE2 and boosts binding to S protein, while rs191860450 (p.Ile468Val), which is more prevalent in Asians, may alter the ACE2–S protein interaction characteristics, but the significance of this is unclear.^183,194^ The variant p.Arg514Gly, located in the AGT–ACE2 interaction surface, was predicted to increase COVID-19 risk by altering RAAS function.^171^ The higher COVID-19 mortality in Italy may be partly explained by the role of rs41303171 (p.Asn720Asp), which is more prevalently carried by Italians, in promoting TMPRSS2 cleaving and viral intake.^195^

The variants rs73635825 (p.Ser19Pro) and rs766996587 (p.Met82Ile) exclusively presented in Africans may reduce encoded protein stability and binding affinity to S protein binding sites.^4,8,196^ The European-specific variant rs1448326240 (p.Glu239His) is thought to be an interaction-inhibiting variant and lead to a lower SARS-CoV-2 susceptibility.^184^ Rs143936283 (p.Glu329Gly) has a lower binding affinity for the S protein, implying that rs143936283 may confer a lower probability of viral attachment and some level of resistance against infection.^195^ The variants p.Leu351Val and rs762890235 (p.Pro389His), which occur in the ACE2–S protein interaction region, are predicted to interfere with the internalization process.^197^ Rs961360700 (p.Asp355Asn) and rs1396769231 (p.Met383Thr) were also predicted to adversely affect ACE2 stability.^196^ The four variants located in the ACE2 dimeric interface, p.Arg708Trp, p.Arg710Cys, p.Arg710His, and p.Arg716Cys, could affect ACE2 cleavage by TMPRSS2 and change the dimer formation, which may be responsible for the milder COVID-19 symptoms in Europeans having these four variants.^171^

Heterogeneous *ACE2* expression in different ethnic groups might be a measure of differential population reactions to COVID-19. For example, Asians have a higher *ACE2* expression than African Americans and Caucasians. The expression quantitative loci for upregulating *ACE2* can be up to almost 100% in East Asians, which are over 30% higher than other racial groups.^185,198^ The prevalence of *ACE2*-downregulating variants is 54% in non-Finnish Europeans, 39% in Africans/African Americans, and 2–10% in Latinos/admixed Americans, East Asians, Finns, and South Asians, while Amish and Ashkenazi Jewish populations seem to carry none of such variants.^171^ Highly penetrant dominant trait presenting in the *ACE2* gene probably affects familial clusters.^10^ Approximately 320–365 out of every 100,000 humans possess SNVs decreasing spike binding, while 4–12 of every 100,000 humans possess SNVs increasing spike binding. Specific SNVs affecting S protein binding are more abundant in individuals of a certain ancestry, and this frequency may vary six-fold between different ancestries.^199^

Furthermore, the *ACE2* gene, mapped at the pseudoautosomal regions of X-chromosome, could escape XCI more probably, which likely confers females a double ACE2 dosage to compensate for the loss of membrane ACE2 due to SARS-CoV-2.^175,178^ One study showed that, in the hemizygous state, >50% of the variants probably influence the binding of the human ACE2 and the viral S1 protein.^184^ *ACE2* interaction-booster and interaction-inhibitor variants can be more significant in males and the former may result in a higher mortality rate in males than females.^100^ The fact that the *ACE2* gene expression could be elevated in females due to a skewed XCI, providing a larger ACE2 pool to maintain the fundamental balance of RAAS-regulatory axis in multiple organs after viral infection, could partly explain the lower frequency of severe COVID-19 in females than males.^17,172^

### Xq12 and the androgen receptor gene (*AR*)

The 15-bp *AR* binding element is the critical part of the *TMPRSS2* promoter for androgen’s binding and its transcription regulation.^200,201^ This *AR* element is a polymorphic unit with CAG trinucleotide repeat length variations in the *AR* gene’s first exon.^202^ Length variation can determine both transcriptional activity and androgen resistance strength that shorter CAG repeat polymorphism may lead to *TMPRSS2* overexpression and link to androgen sensitivity, as well as more severe COVID-19.^176^ The variation of greater COVID-19 mortality in males than females or infants of either gender may be explained by androgen-mediated *ACE2* and *TMPRSS2* expressions.^101,200,203^ Ethnicity-based vulnerability may be explained by the *AR* gene polymorphisms that African-American males with shorter CAG repeat lengths have a disproportionate mortality rate than non-Hispanic white males.^201,202^

## Conclusions and perspectives

The world is still suffering from the COVID-19 outbreak and the ultimate outcomes are, so far, unmeasurable, but the global economic, social, and political disruptions caused by this pandemic are poised to worsen in the foreseeable future.^11,204^ Therefore, understanding the causal relationships between host genetic basis and COVID-19 is urgently needed to identify biomarkers for individuals at high risk, which might also provide potential targets for therapy.^6,98^ Focusing on these variants, which relate to disease susceptibility and severity through viral trafficking pathways or drug curative effect, could better identify risk subjects and effectively control the disease. Large data consortiums are organizing to produce, share, and analyze data, such as the COVID19 Host Genetics Initiative and the COVID Human Genetic Effort.^12,205^ As more host genetic factors associated with COVID-19 are identified, it should become possible to create tests that would predict the susceptible populations and allow for classifying and safeguarding them.^190^ In summary, we extract and review positive results from vast reported papers. However, for a certain variant, large-scale meta-analyses combining data from multiple consistent studies with reliable statistical significance would be helpful to find therapy targets.

Ongoing investigations into COVID-19 and individual genetic makeup are fueling global research to develop vaccines, prioritize individuals for treatment, and discover potential drug target candidates.^95^ The antiviral drug Veklury (remdesivir) is the first and the only treatment for COVID-19 approved by the US Food and Drug Administration (https://www.fda.gov). Given that remdesivir is a broad-spectrum antiviral drug, better direct-target-based antiviral therapies that intervene in SARS-CoV-2-infected pathways are anticipated.^206^ Human genetic basis of COVID-19, which is well known to impact disease susceptibility and severity, may offer novel insights into COVID-19 therapies and controls through identifying particular genes and pathways. Large-scale screening of potential targeted drugs and experimental therapeutic studies would be helpful to develop new drugs or discover repurposing opportunities for existing drugs.^148^ In order to end this century nightmare early, when it comes to ethical considerations and/or societal questions, further investigations should pay attention to (1) ensuring the validity and usefulness of the reported studies, (2) not undermining the necessity of solidarity in the public health action, (3) not affecting individuals’ action ability or making them be discrimination targets, and (4) perfecting genetic information-related legislation.^207^

## Supplementary information

Supplementary materials
