# Supplementary material for: Human genetic basis of coronavirus disease 2019
Source: Signal Transduct Target Ther. 2021 Sep 20;6:344. doi: 10.1038/s41392-021-00736-8 (PMC8450706; doi:10.1038/s41392-021-00736-8)
Supplement: Supplementary file 1 — Supplementary materials [file 41392_2021_736_MOESM1_ESM.pdf]

Supplementary Materials for

Human genetic basis of coronavirus disease 2019

Hao Deng, Xue Yan, Lamei Yuan

Correspondence to: hdeng008@yahoo.com

This PDF file includes:

Figures. S1 to S2

Figure. S1

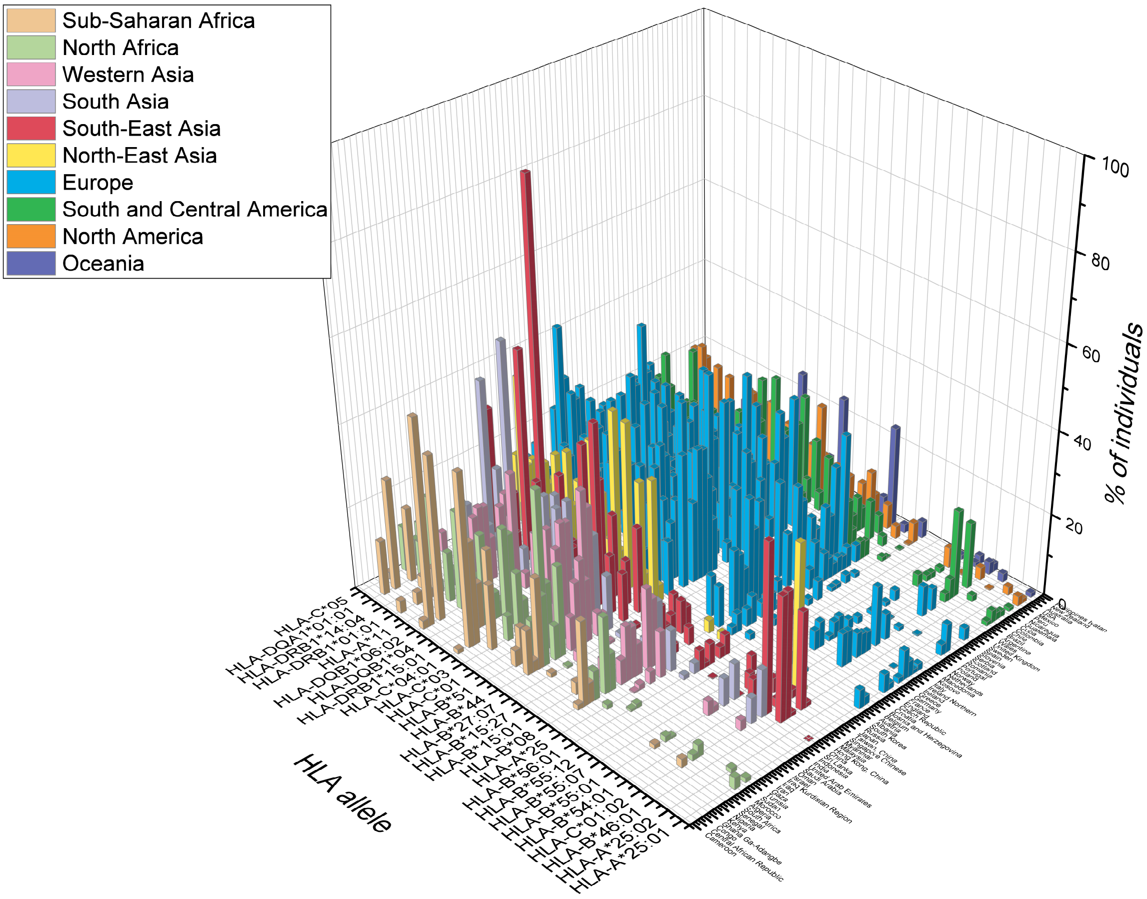

Fig. S1. Predicted-risk HLA allele frequencies in global regions. Note: the data come from the Allele Frequency Net Database (http://www.allelefrequencies.net/hla.asp, updated on June 1, 2021). If one region has more than one result with different studies, the largest one is used.

Figure S2

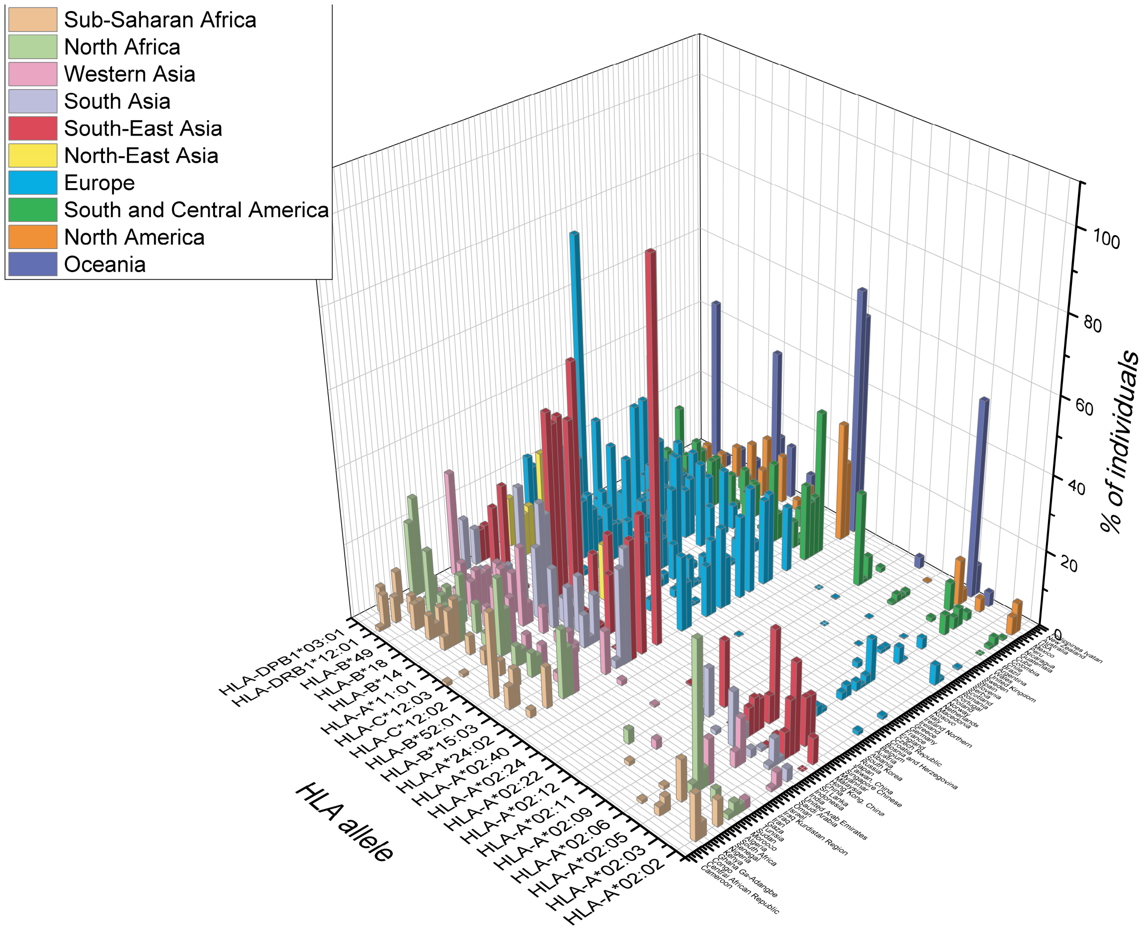

Fig. S2. Predicted-protective HLA allele frequencies in global regions. Note: the data come from the Allele Frequency Net Database (http://www.allelefrequencies.net/hla.asp, updated on June 1, 2021). If one region has more than one result with different studies, the largest one is used.
